# Generalized Laminar Population Analysis (gLPA) for Interpretation of Multielectrode Data from Cortex

**DOI:** 10.3389/fninf.2016.00001

**Published:** 2016-01-25

**Authors:** Helena T. Głąbska, Eivind Norheim, Anna Devor, Anders M. Dale, Gaute T. Einevoll, Daniel K. Wójcik

**Affiliations:** ^1^Laboratory of Neuroinformatics, Department of Neurophysiology, Nencki Institute of Experimental Biology of the Polish Academy of SciencesWarsaw, Poland; ^2^Department of Mathematical Sciences and Technology, Norwegian University of Life SciencesAas, Norway; ^3^Departments of Neurosciences and Radiology, University of CaliforniaSan Diego, La Jolla, CA, USA; ^4^Martinos Center for Biomedical Imaging, Harvard Medical School, Massachusetts General HospitalCharlestown, MA, USA; ^5^Department of Physics, University of OsloOslo, Norway

**Keywords:** LFP analysis, local field potential (LFP), model-based analysis, signal decomposition, computational neuroscience, multi-unit activity, MUA, thalamocortical

## Abstract

Laminar population analysis (LPA) is a method for analysis of electrical data recorded by linear multielectrodes passing through all lamina of cortex. Like principal components analysis (PCA) and independent components analysis (ICA), LPA offers a way to decompose the data into contributions from separate cortical populations. However, instead of using purely mathematical assumptions in the decomposition, LPA is based on physiological constraints, i.e., that the observed LFP (low-frequency part of signal) is driven by action-potential firing as observed in the MUA (multi-unit activity; high-frequency part of the signal). In the presently developed generalized laminar population analysis (gLPA) the set of basis functions accounting for the LFP data is extended compared to the original LPA, thus allowing for a better fit of the model to experimental data. This enhances the risk for overfitting, however, and we therefore tested various versions of gLPA on virtual LFP data in which we knew the ground truth. These synthetic data were generated by biophysical forward-modeling of electrical signals from network activity in the comprehensive, and well-known, thalamocortical network model developed by Traub and coworkers. The results for the Traub model imply that while the laminar components extracted by the original LPA method overall are in fair agreement with the ground-truth laminar components, the results may be improved by use of gLPA method with two (gLPA-2) or even three (gLPA-3) postsynaptic LFP kernels per laminar population.

## 1. Introduction

The rapid development of silicon-based multielectrodes allowing for simultaneous recording of extracellular potentials at tens or hundreds of contacts in an *in vivo* setting, has revived the interest in the local field potential (LFP; Bedárd and Destexhe, 2012; Buzsáki et al., 2012; Pettersen et al., 2012; Einevoll et al., 2013a,b). In cortical recordings the LFP, i.e., the low-frequency part (≤ 500 Hz) of the extracellular potential, is thought to largely reflect synaptic input currents and their associated return currents (Pettersen et al., 2008; Einevoll et al., 2013a). While the interpretation of the high-frequency part of the signal (i.e., the multiunit activity, MUA) in terms of spiking activity of neurons surrounding the contacts seems well established (Buzsáki, 2004; Pettersen et al., 2008; Einevoll et al., 2012), an interpretation of the LFP in terms of activity in specific neurons or neuronal populations is much more challenging (Einevoll et al., 2013a).

Current-source density (CSD) analysis has been a standard tool for analysis of LFP (Nicholson and Freeman, 1975; Mitzdorf, 1985; Pettersen et al., 2006; Potworowski et al., 2012; Wójcik, 2015). From simultaneous recordings of LFPs at many different spatial positions, the net volume density of current entering or leaving the extracellular space, can be estimated. Due to the more local nature of CSD compared to the LFP, the CSD may be easier to interpret, but the CSD still does not give direct information regarding what neurons or neural populations are involved in generating the signal. Attempts have therefore been made to decompose the LFP into a set of putative cortical populations components by means of standard mathematical data analysis tools like principal components analysis (PCA; Barth and Di, 1991) and independent components analysis (ICA; Leski et al., 2010; Makarov et al., 2010; Glabska et al., 2014). Dynamical causal modeling (DCM; David and Friston, 2003) represents an alternative approach where neurophysiological data, also LFP (Moran, 2011), is fitted to a set of differential equations describing the dynamics of underlying neural-mass models representing the neural populations.

Laminar population analysis (LPA; Einevoll et al., 2007) takes a different approach and makes physiological, rather than mathematical, assumptions to determine the population decompositions. In particular, the recorded LFP is assumed to be causally generated by the recorded spikes as measured in the MUA. In LPA, the LFP and MUA data are thus jointly modeled. In the orginal application, LPA was applied to stimulus-evoked linear (laminar) multielectrode data from barrel cortex of anesthetized rats following single whisker flicks, and the data was seen to be well accounted for by a model with four cortical populations: one supragranular, one granular and two infragranular populations (Einevoll et al., 2007).

The original LPA method made the assumption of a single spatiotemporally separable LFP kernel following population firing in each laminar population. In the present *generalized laminar population analysis (gLPA)* we go beyond this and allow for several independent postsynaptic LFP kernels per population, each kernel consisting of a spatial (depth) profile multiplied by a temporal kernel. The physical justification for multiple kernels is that (i) given the multiple time scales involved in synaptic activation and effects on the postsynaptic cells and (ii) the different biophysical properties and morphologies in the different postsynaptic cells, one cannot expect a priori a single spatiotemporally separable kernel to fully account for the LFP induced by action-potential firing even in a single neural population. Further, there may also be LFP contributions associated with the spike signature itself (Buzsáki et al., 2012; Schomburg et al., 2012).

gLPA involves a larger set of basis functions compared to the original LPA method and will thereby by design fit the LFP data better. More basis functions increase the risk of overfitting, and gLPA is thus tested against benchmarking data set for which the “ground truth” is known. Such benchmarking techniques are well established for evaluation of analysis methods, also in neuroscience (Pettersen et al., 2008; Leski et al., 2011; Denker et al., 2012; Glabska et al., 2014; Hagen et al., 2015; Ness et al., 2015). Here we test the new gLPA method on virtual LFP found from biophysical forward-modeling of electrical signals from network activity in the comprehensive (3500 neurons), and well-known, thalamocortical network model developed by Traub et al. (2005) as modified by Glabska et al. (2014). In this virtual model world the spike times for all neurons are known, facilitating testing of the LFP-modeling part of gLPA before introducing confounding effects from inaccurate estimates of the population firing rates. The results for the Traub model imply that while the laminar components extracted by the original LPA method overall are in fair agreement with the ground-truth laminar components, the results may be improved by use of the gLPA method with two (gLPA-2) or even three (gLPA-3) postsynaptic LFP kernels per laminar population.

## 2. Methods

### 2.1. Generalized laminar population analysis (gLPA)

The (virtual) LFP ϕ to be analyzed were obtained as *N*_*ch*_ × *N*_*t*_ arrays of data where *N*_*ch*_ is the number of laminar electrode channels, and *N*_*t*_ is the number of time points with data.

A general expression for decomposition of a two-dimensional function ϕ(*z*_*i*_, *t*_*j*_) into spatiotemporally separable components reads:

(1) $$\begin{array}{l} \phi \left(z_{i},t_{j}\right)=\underset{n}{\sum }f_{n}\left(z_{i}\right)g_{n}\left(t_{j}\right)\;\;\;. \end{array}$$

Here *z*_*i*_ denotes the depth of the electrode contact (*i* = 1, …, *N*_*ch*_), and *t*_*j*_ = *j*Δ*t* where *j* = 1, …, *N*_*t*_.

In *principal component analysis (PCA)*, for example, the expansion functions *f*_*n*_(*z*_*i*_) (called spatial loadings) and *g*_*n*_(*t*_*j*_) (called temporal scores) are chosen so that the first component picks up most of the data variance, the second component most of the remaining variance, etc. (Gershenfeld, 1999).

The key idea of *LPA*, both the original (Einevoll et al., 2007) and the present generalized version, is that that the observed LFP is driven by the observed population firing *r*_*n*_(*t*). In Einevoll et al. (2007) the population firing rates *r*_*n*_(*t*_*j*_) were obtained from fitting of the experimental MUA data. In the present study the data is model-based and we know all the spike times. Therefore, in the majority of results (Sections 3.1–3.3) we find *r*_*n*_(*t*_*j*_) directly from summing over the spikes of all excitatory neurons from layer *n* (as described below). Only in Section 3.4 we check how the estimation of firing rates from measurements affects the results of gLPA.

In the original LPA (later referred to as *gLPA-1* for reasons explained below) we used the following decomposition (Einevoll et al., 2007):

(2) $$\begin{array}{l} \phi_{\text{gLPA-1}}^{m}\left(z_{i},t_{j}\right)=\overset{N_{\text{pop}}}{\underset{n=1}{\sum }}L_{n}\left(z_{i}\right)\left(h⊗r_{n}\right)\left(t_{j}\right)\;. \end{array}$$

Here (*h* ⊗ *r*_*n*_)(*t*_*j*_) is the temporal convolution given by

(3) $$\begin{array}{l} \left(h⊗r_{n}\right)\left(t_{j}\right)=\overset{\infty }{\underset{j^{\prime }=-\infty }{\sum }}h\left(t_{j^{\prime }}\right)r_{n}\left(t_{j}-t_{j^{\prime }}\right)\;, \end{array}$$

where $t_{j^{\prime }}=j^{\prime }\Delta t$.

In the new generalized LPA (gLPA) we assume the following generalized form:

(4) $$\begin{array}{l} \phi_{\text{gLPA-K}}^{m}\left(z_{i},t_{j}\right)=\overset{N_{\text{pop}}}{\underset{n=1}{\sum }}\overset{K}{\underset{k=1}{\sum }}L_{n}^{k}\left(z_{i}\right)\left(h^{k}⊗r_{n}\right)\left(t_{j}\right)\;. \end{array}$$

This implies that (unless *K* = 1) the contribution to the LFP from each population is no longer factorizable into a spatial and a temporal component. The physical interpretation is that (i) given the multiple time scales involved in synaptic activations and (ii) the different biophysical properties and morphologies in the different postsynaptic cells, one cannot expect a priori a single spatiotemporally separable kernel to fully account for the LFP induced by action-potential firing even in a single neural population. This is reminiscent of the non-product nature of the LFP and CSD generated by individual cell populations which we reported in Glabska et al. (2014). Rather, the postsynaptic contribution to the LFP induced by firing in each population *n* may be described more adequately by *K* distinct components with different spatial ($L_{n}^{k}$) and temporal (*h*^*k*^) profiles. One or more of the components could, for example, describe the LFP following synaptic activation of the postsynaptic population (identified as the main contribution for gLPA-1 in Einevoll et al., 2007), while others could be LFP associated with the extracellular signature of the spiking itself, for example, due to spike afterhyperpolarization.

In the present paper we investigate several variations of gLPA with different number of components *K*, and refer to the various versions as gLPA-K. Thus, the orginal LPA version is labeled gLPA-1, while a version with two LFP components for each presynaptic population *n* is referred to as gLPA-2, and so on.

For convenience, we introduce new auxiliary variables for the time-convolved firing rates:

(5) $$\begin{array}{l} R_{n}^{k}\left(t\right)=\left(h^{k}⊗r_{n}\right)\left(t\right), \end{array}$$

so that Equation (4) can be written more compactly as

(6) $$\begin{array}{l} \phi_{\text{gLPA-K}}^{m}\left(z_{i},t_{j}\right)=\overset{N_{\text{pop}}}{\underset{n=1}{\sum }}\overset{K}{\underset{k=1}{\sum }}L_{n}^{k}\left(z_{i}\right)R_{n}^{k}\left(t_{j}\right)\;. \end{array}$$

In the original LPA (gLPA-1) (Einevoll et al., 2007) the temporal kernel was assumed to be a (normalized) exponentially decaying function of the form

(7) $$\begin{array}{l} h\left(t_{j};\tau ,\Delta \right)=\frac{1}{\tau }e^{-\left(t_{j}-\Delta \right)∕\tau }\Theta \left(t_{j}-\Delta \right)\;\;\;. \end{array}$$

where Θ(*t*_*j*_) is the Heaviside unit step function [Θ(*t*_*j*_ ≥ 0) = 1, Θ(*t*_*j*_ < 0) = 0], and τ and Δ are the time constant and a time-delay parameter, respectively.

In gLPA-K (at least in the present application) we still use exponential kernels, but we now have different values for both the time constant and time-delay parameter for the *K* kernels specified by τ^*k*^ and Δ^*k*^ (*k* = 1, …, *K*), giving a total of 2*K* parameters to be fitted against experiment. Note that one could also make these time constants and time-delay parameters different for each population *n* at the expense of adding many new parameters to be determined when fitting the data.

### 2.2. Generation of virtual LFP data from network model

To investigate the quality of the decomposition given by Equation (4), as well as the interpretation of individual terms, we used simulated data. The data were generated with a single-column model for thalamocortical system published by Traub et al. (2005). The model contains 3560 multicompartment cells from four cortical layers (layer 2/3, 4, 5, and 6) and the thalamus as described in Table 1. The somas of each population were randomly placed in cylindrical boxes with radii of 200 μm and vertical extensions as described in Table 2. More details of the employed network model can be found in Glabska et al. (2014).

**Table 1:** **Cell types used in the model, numbers of sections in each cell, and numbers of cells in each population, cf. Glabska et al. (2014)**.

**Soma location**	**Population name**	**Number of sections**	**Number of cells**
Layer 2/3	Pyramidal regular spiking (RS)	74	1000
Layer 2/3	Pyramidal fast rythmic bursting (FRB)	74	50
Layer 2/3	Superficial interneurons—basket (BASK), axoaxonic (AX), low-threshold spiking (LTS)	50	3 × 90
Layer 4	Spiny stellate (SS)	59	240
Layer 5	Pyramidal tufted intrinsic bursting (IB)	61	800
Layer 5	Pyramidal tufted regular spiking (RS)	61	200
Layer 5/6	Deep interneurons—basket (BASK), axoaxonic (AX), low-threshold spiking (LTS)	59	3 × 100
Layer 6	Pyramidal non-tufted regular spiking (RS)	59	500
Thalamus	Thalamocortical relay (TCR)	139	100
Thalamus	Reticular nucleus (NRT)	59	100
Total			3560

**Table 2:** **Layer organization of cortical column model showing the layer-depth intervals of the soma positions of the neuronal populations**.

**Layer**	**Layer depth (μm)**	**Model populations**	**gLPA pop. no**.
2/3	450–850	**RS**, **FRB**, BASK, AX, LTS	1
4	850–1150	**SS**	2
5	1150–1650	**IB**, **RS**, BASK, AX, LTS	3
6	1650–2150	**RS**, BASK, AX, LTS	4

Cortical depths are measured compared to a reference point (i.e., zero depth) set 450 μm above the top of the layer containing the somas of the layer 2/3 cells, cf. Glabska et al. (2014). Spikes from neurons in the populations shown in bold are used to compute the laminar population firing rates used in gLPA. gLPA laminar population numbers are listed in the rightmost column.

**Figure 1:**
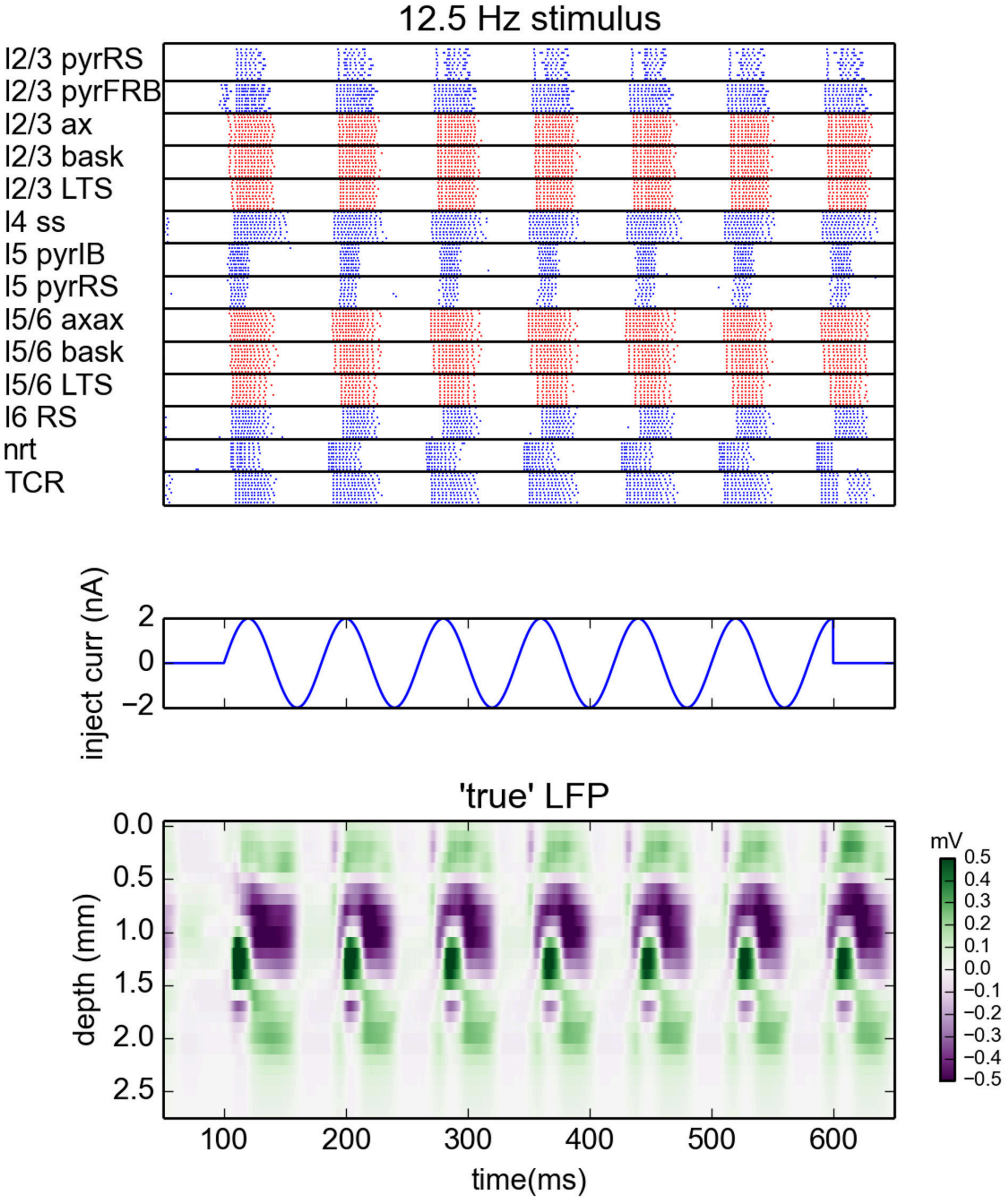
**Generation of virtual LFP data**. **(Top)** Raster plot of the network activity for the simulation with oscillatory current with frequency 12.5 Hz injected to the thalamus for times between 100 and 600 ms **(Middle)**. Blue dots indicate spikes of excitatory neurons, red dots indicate spikes of inhibitory cells. For figure clarity, only activity of 10 randomly selected cells from every population is shown (representative of the populations). Layer 5 and layer 6 pyramidal cells were additionally depolarized by injecting 1 and 0.75 nA currents into their somas, see Traub et al. (2005). **(Bottom)** Depth-resolved LFP generated by the network model in the same time period.

The simulations were run with the NEURON 7.3 simulator (Hines and Carnevale, 1997). The network was driven by sinusoidal currents (amplitude of 2 nA) injected into somas of all thalamocortical relay cells (TCR). For each stimulation frequency the network was simulated for 650 ms. The sinusoidal current stimulus was turned on 100 ms after the start of the simulation and lasted for 500 ms, cf. middle panel in Figure 1. This gives oscillatory firing responses both in the thalamic and cortical populations (Figure 1, top panel). This simulation procedure was repeated for a set of eight different stimulus frequencies, i.e., 2, 4, 8, 12.5, 25, 50, 100, and 200 Hz.

Following well-established volume conductor theory, the extracellular potentials were computed by summing over weighted contributions from all transmembrane currents in the vicinity of the (virtual) recording electrode (Rall and Shepherd, 1968; Holt and Koch, 1999; Lindén et al., 2014). Here we assumed a homogeneous, isotropic and Ohmic extracellular medium so that the extracellular potential ϕ at a position **r** in the presently used *point-source approximation* (Lindén et al., 2014) can be computed according to the formula:

(8) $$\phi (r,\;t)=\frac{1}{4\pi \sigma }\sum_{n}\frac{I_{n}(t)}{\left|r-r_{n}\right|}.$$

The sum here goes over all compartments of all neurons in the model network, and *I*_*n*_ is the transmembrane current from the *n*-th current source positioned at **r**_n_. Further, σ is the extracellular conductivity, here assumed to have the value σ = 0.3 S/m.

To mimic the data recorded by linear (laminar) multielectrodes in the typical *in vivo* setting (e.g., Einevoll et al., 2007), we computed the extracelluar potentials at a set of equidistant contact positions (100 μm intercontact distance) along the cylindrical axis of the cortical model network using the point-electrode approximation where effects from the physical extention of the metal contact are neglected (Lindén et al., 2014; Ness et al., 2015). Extracellular potentials ϕ(**r**, *t*) were computed and stored with a time resolution of 0.1 ms. To obtain the LFP the potential was low-pass filtered below 100 Hz with a second-order Butterworth filter and decimated to give a time resolution of 0.5 ms. The computation of the extracellular potentials from the transmembrane currents extracted from the NEURON simulation, as well as all postprocessing, was done in Python and MATLAB.

In addition to the full network simulations of spikes and LFPs, we also did separate LFP calculations for a disconnected network driven by spiking activity from a full simulation, to probe the accuracy and applicability of gLPA. We first recorded the spike times driving every cell in the simulation of fully connected network. Then the spike times originating from a particular population of neurons were used to generate synaptic inputs onto their normal postsynaptic targets in an otherwise disconnected network. Therefore, in this situation the computed LFPs stem only from transmembrane currents in the postsynaptic target neurons of the presynaptic population of interest.

### 2.3. Estimation of population firing rates

Implementation of generalized LPA, just like the original LPA (Einevoll et al., 2007), consists of two steps. One first needs to estimate the firing rates of cell populations from individual layers. Then one needs to estimate the parameters of the kernel *h*^*k*^ and spatial profiles $L_{n}^{k}$.

#### 2.3.1. Population firing rates from ground-truth spike times

In Einevoll et al. (2007) the population firing rates *r*_*n*_(*t*_*j*_) were obtained from the MUA, that is, the high-frequency part of the recorded extracellular potentials. Here we know all the spike times, and so in the majority of results (Sections 3.1–3.3) we find *r*_*n*_(*t*_*j*_) directly from summing over the spikes of all excitatory neurons in a layer. In the present network model, four distinct layers are considered, i.e., layer 2/3, layer 4, layer 5, and layer 6, cf. Tables 1–2. We thus consider four (*N*_pop_ = 4) laminar populations in our gLPA analysis as in Einevoll et al. (2007), cf. Table 2. For the layer-2/3 laminar population (*n* = 1) the population firing rate *r*_1_(*t*) is found by summing all spikes of the layer-2/3 RS neurons and the layer-2/3 FRB neurons (cf. Table 1) using binning intervals of 0.5 ms. For the layer-4 laminar population (*n* = 2) the firing rate *r*_2_(*t*) is correspondingly found by summing over all layer 4 SS neurons. For the layer-5 laminar population (*n* = 3) the pyramidal IB and RS neurons are summed over to get the firing rate *r*_3_(*t*), while the layer-6 laminar population firing rate *r*_4_(*t*) is found by summing over the pyramidal non-tufted RS cells.

#### 2.3.2. Population firing rates from (virtual) MUA

In Section 3.4 we study the application of gLPA when population firing rates are not *a priori* known, but rather must be estimated the high-frequency part of the generated extracellular potentials, i.e., the MUA (multi-unit activity). To obtain the MUA we applied to ϕ the same procedure to the virtual electrical data (cf. Section 2.2) as in the original LPA paper (Einevoll et al., 2007), that is, ϕ was filtered between 750 and 5000 Hz using a zero phase-shift second-order Butterworth filter and rectified along the time axis and decimated by a factor 10 along the time axis to provide the same time resolution as the LFP data. In the estimation of the population firing rates from MUA data, the same procedure as in the original LPA paper (Einevoll et al., 2007) is used: The MUA data is decomposed into a sum over spatiotemporally separable contributions from several neuronal populations

(9) $$\begin{array}{l} \phi_{MUA}\left(z_{i},t_{j}\right)=\overset{N_{pop}}{\underset{n=1}{\sum }}M_{n}\left(z_{i}\right)r_{n}\left(t_{j}\right), \end{array}$$

where *M*_*n*_(*z*_*i*_) is a MUA spatial depth profile for action-potential firing in population *n*. This profile will depend on the spatial spread of the extracelluar spike of the individual neurons in the population as well as the distribution of their spatial positions in the cortical lamina. As in Einevoll et al. (2007) we assumed trapezoidal forms of *M*_*n*_(*z*_*i*_) specified by three parameters. These parameters were fitted to minimize the mean square difference between the decomposition assumed in Equation 9 and the (virtual) MUA recordings, see Appendix 1 in Einevoll et al. (2007) for details.

### 2.4. Estimation of laminar LFP components in gLPA

The second step of fitting the gLPA-models to the LFP data given the population firing rates *r*_*n*_(*t*), was done largely as described in Appendix 1 of Einevoll et al. (2007). Given the population firing rates *r*_*n*_(*t*), the time constants τ and delays Δ of the temporal kernels and the LFP profiles were fitted to the virtual LFP data (Note that in the case where the population firing rates were found from fitting to the MUA data, a better procedure could have been to fit the model to the MUA and LFP data in a single integrated step, rather than in the present simpler two-step procedure. However, we used the same method as in the original LPA paper, Einevoll et al., 2007).

The concatenated virtual LFP data from all eight data sets with sinusoidal stimulation was used in the analysis to fit a single set of model parameters (Δ's, τ's and laminar LFP profiles). As in Einevoll et al. (2007) the mean LFP prior to stimulus onset was taken as a baseline and subtracted from the raw LFP before application of gLPA. This baseline was computed and subtracted for each channel separately, with the baseline computed as the mean LFP on the interval between 50 and 100 ms after the start of the simulation.

As in Einevoll et al. (2007) the relative mean square deviation between the LFP predicted by gLPA and the “true” (ground-truth) LFP is used as error measure, i.e.,

(10) $$e_{L}=\frac{\sum_{i\;=\;1}^{N_{ch}}\sum_{j\;=\;1}^{N_{t}}{\left(\phi (z_{i},t_{j})-\phi_{gLPA}^{m}(z_{i},t_{j})\right)}^{2}}{\sum_{i\;=\;1}^{N_{ch}}\sum_{j\;=\;1}^{N_{t}}{\left(\phi (z_{i},t_{j})\right)}^{2}}\;,$$

where ϕ is the “true” LFP data and $\phi_{gLPA}^{m}$ the gLPA prediction. Here *N*_*ch*_ = 28 is the number of virtual recording channels (contacts), and *N*_*t*_ is the number of time events (0.5 ms between each). Unlike in Einevoll et al. (2007), the de solver (differential evolution algorithm) was used in the fitting procedure to minimize the error (downloaded from openopt.org). In the optimization the upper bounds of the allowed values of the delay and time constants were set to: (i) gLPA-1: $\Delta_{1}^{\text{max}}=50$ ms, $\tau_{1}^{\text{max}}=10$ ms, (ii) gLPA-2: $\Delta_{1}^{\text{max}}=50$ ms, $\tau_{1}^{\text{max}}=\;10$ ms, $\Delta_{2}^{\text{max}}=100$ ms, $\tau_{2}^{\text{max}}=300$ ms, (iii) gLPA-3: $\Delta_{1}^{\text{max}}=50$ ms, $\tau_{1}^{\text{max}}=10$ ms, $\Delta_{2}^{\text{max}}=50$ ms, $\tau_{2}^{\text{max}}=10$ ms, $\Delta_{3}^{\text{max}}=100$ ms, $\tau_{3}^{\text{max}}=300$ ms. Lower bounds were always zero.

### 2.5. CSD analysis

We also analyzed the resulting LPA laminar LFP profiles by means of CSD analysis (Nicholson and Freeman, 1975; Mitzdorf, 1985; Pettersen et al., 2006; Potworowski et al., 2012; Wójcik, 2015). The *kCSD*-method (Potworowski et al., 2012) was used assuming a cylindrical CSD column with a radius of 0.4 mm, which takes into account the region covering the dendrites protruding from all the cells in the column. Note that the radius assumed in reconstruction did not affect the results significantly as observed in Potworowski et al. (2012).

## 3. Results

### 3.1. Virtual data from network simulations

While concatenated model data resulting from use of the full stimulus protocol (i.e., the full set of oscillatory thalamic input outlined in Methods) was used in the gLPA analysis, we will, for presentational clarity in the following, focus on the results for the case with 12.5-Hz oscillatory thalamic input. The spike raster plots and corresponding (virtual) LFP data for the full 500 ms period when the thalamic input is on, are shown in Figure 1. Generally, the responses to each of the seven depicted synaptic input volleys look similar. However, the response to the first volley is somewhat different due to transient network activation effects, while the response to the last volley is different due to the abrupt abortion of thalamic input at 600 ms in our simulation.

In the following we will illustrate the outcome of the LPA analysis by showing results from the sixth response volley, i.e., from *t* = 500 to 580 ms, where transient network activation effects are clearly absent. Note, however, that the LPA analysis is based on the full data set, as are the reported error measures.

### 3.2. Results from laminar population analysis

Here we first describe results from using the original LPA method (gLPA-1, Einevoll et al., 2007), and then move on to investigate the situation with two (gLPA-2) or three (gLPA-3) separate postsynaptic LFP components for each laminar population. As in Einevoll et al. (2007), in all cases we assume four laminar populations, i.e., *N*_*pop*_ = 4, cf. Table 2.

#### 3.2.1. gLPA-1: one postsynaptic LFP profile

**Figure 2:**
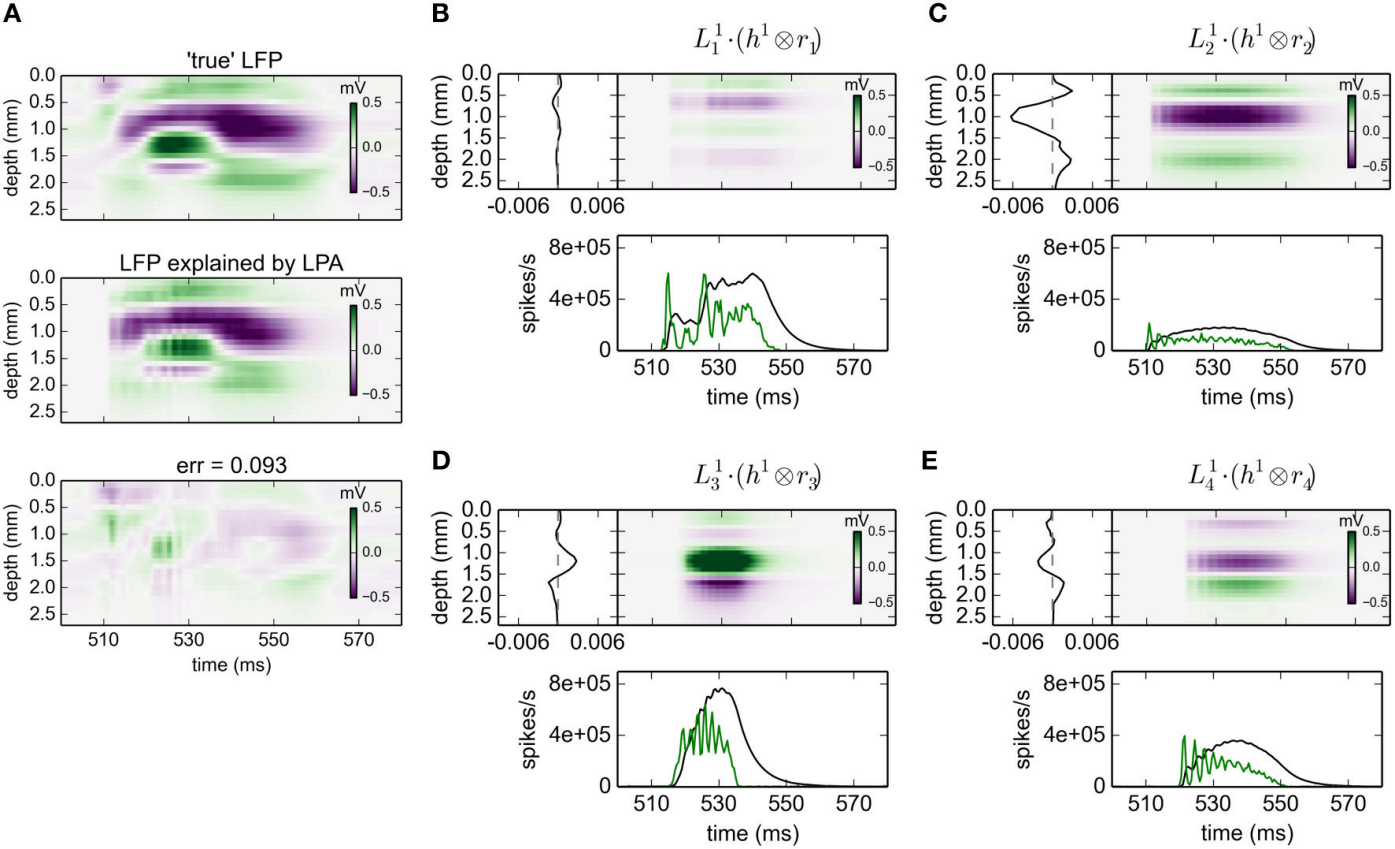
**gLPA-1 decomposition of LFP data**. Results from fitting gLPA-1 (*K* = 1; Equation 2) to LFP data for a time segment where the network is driven by 12.5 Hz oscillatory input in the thalamic relay cells. **(A)** Comparison of ground-truth LFP (“true” LFP; top) with LPA prediction (middle) with difference shown in bottom panel. **(B–E)** Estimated contributions from the four laminar components (color plots) with spatial profiles ($L_{n}^{1}$) shown left and temporal profiles [green:*r*_*n*_(*t*); black:$R_{n}\left(t\right)=h^{1}⊗r_{n}$] below. Fitted parameters: Δ_1_ = 0.40 ms, τ_1_ = 5.32 ms.

The results for gLPA-1, i.e., the original LPA method, for an 80 ms excerpt of the data are shown in Figure 2. Visual inspection of Figure 2A reveals that the fitted LPA-model results (middle panel) is quite similar to the “true LFP” (top panel), that is, the computed virtual LFP data used as basis for the LPA analysis. The overall relative error for the full data set is 0.093 (while the relative error for the depicted time window shown in the lower panel is 0.095).

The four identified LPA components are shown in Figures 2B–E. The curves in the panels below the 2D plots show both the population firing rates *r*_*n*_(*t*), and the time profile of the postsynaptic LFP contribution $R_{n}^{1}\left(t\right)$ found by convolution of the firing rate with the fitted exponential kernel *h*^1^(*t*) (Equation 5). As seen in these panels, the jaggedness of the population firing rates (*r*_*n*_(*t*)) is largely smoothed out in LFP time profiles ($R_{n}^{1}\left(t\right)=h^{1}⊗r_{n}$) due to the low-pass filtering properties of the convolution operation.

The panels to the left of the 2D plots depict the corresponding spatial LFP profiles $L_{n}^{1}$. Not only are the shapes different, also the amplitudes are seen to vary substantially between the laminar components. Firing of an action potential by a neuron in the L4 population (*n* = 2) is, for example, predicted to give about a five times larger LFP signal compared to firing of a neuron in the L23 population (*n* = 1).

#### 3.2.2. gLPA-2: two postsynaptic LFP profiles

**Figure 3:**
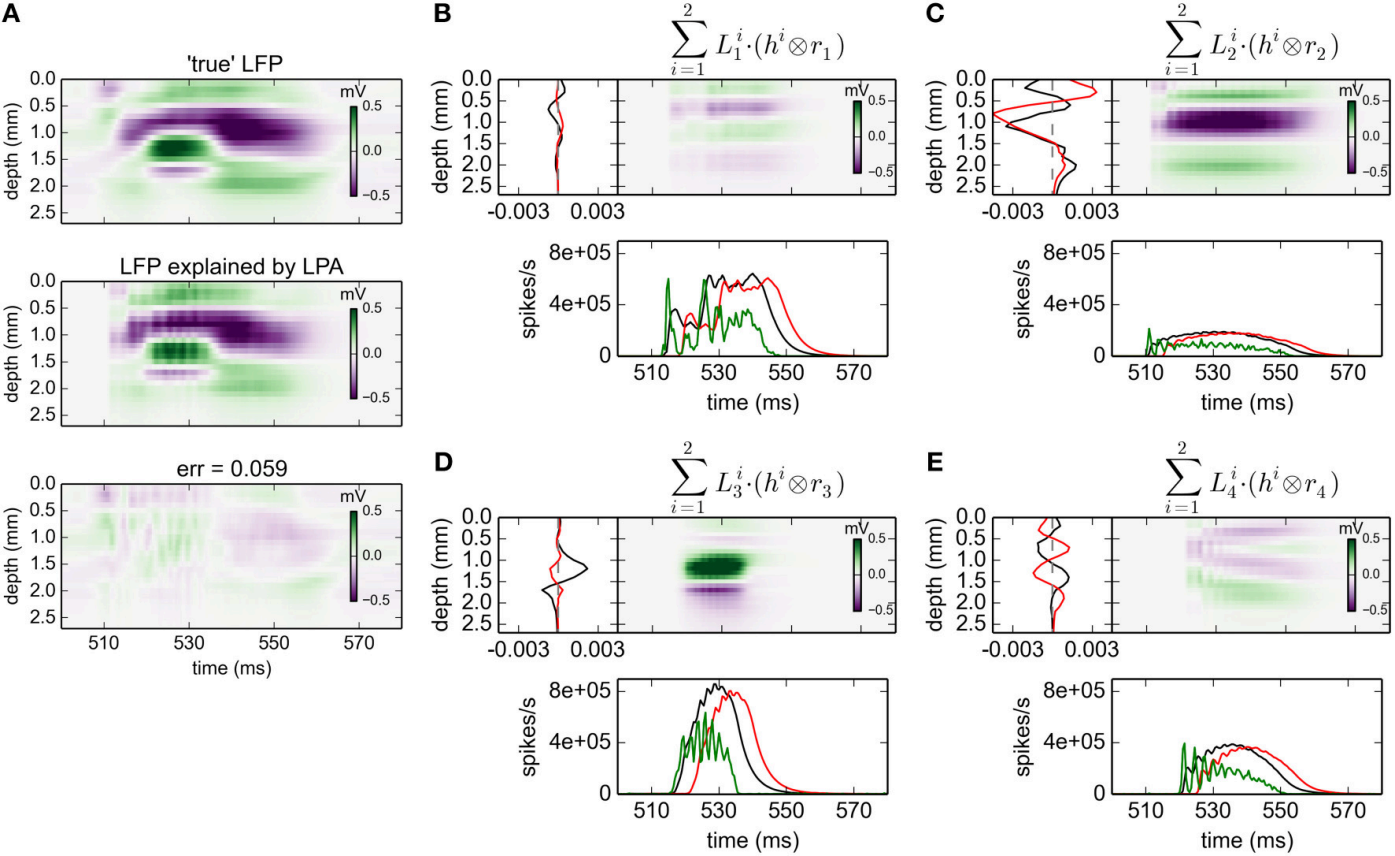
**gLPA-2 decomposition of LFP data**. Results from fitting gLPA-2 (*K* = 2; Equation 4) to LFP data for a time segment where the network is driven by 12.5 Hz oscillatory input in the thalamic relay cells. **(A)** Comparison of ground-truth LFP (“true” LFP; top) with LPA prediction (middle) with difference shown in bottom panel. **(B–E)** Estimated contributions from the four laminar components (color plots) with spatial profiles (black:$L_{n}^{1}$, red:$L_{n}^{2}$) shown left and temporal profiles [green:*r*_*n*_(*t*); black:$R_{n}^{1}\left(t\right)=h^{1}⊗r_{n}$, red:$R_{n}^{2}\left(t\right)=h^{2}⊗r_{n}$] below. Fitted parameters: Δ_1_ = 0.48 ms, τ_1_ = 3.85 ms, Δ_2_ = 4.80 ms, τ_2_ = 4.15 ms.

The corresponding results for gLPA-2 (*K* = 2), i.e., with two postsynaptic LFP profiles assumed for each laminar population, are given in Figure 3. With two LFP postsynaptic profiles (and twice as many free parameters to vary) the relative error is reduced to 0.059 (0.062 for the depicted time window). The associated component profiles in Figures 3B–E reveal that gLPA-2 in particular appears to better account for the LFPs at the onset of strong synaptic activation, for example, around *t* = 510 ms for the LFP generated by the L4 population (*n* = 2) and around *t* = 520 ms for the LFP generated by the L5 population (*n* = 3). For example, unlike gLPA-2, gLPA-1 fails to account for the initial superficial LFP negativity (for depths down to ~0.5 mm) and the simultaneous LFP positivity immediately below (for depths between ~0.5 and ~0.8 mm) for the onset at ~510 ms. This reflects that while gLPA-1 assumes strictly spatiotemporally separable postsynaptic LFP contributions, gLPA-2 does not (since it involves a sum over two spatiotemporally separable contributions). With a sharp onset of strong synaptic input such violation of spatiotemporal separability is expected due to the so called intrinsic dendritic filtering effect (Pettersen and Einevoll, 2008; Lindén et al., 2010; Pettersen et al., 2012). As the high-frequency parts of the LFP will stem from shorter current dipoles than the low-frequency parts, the LFP immediately after the sharp onset of synaptic input will have a different spatial profile than later. This lack of spatiotemporal separability in the LFP from individual populations was also observed in previous applications of the current network model (Glabska et al., 2014), as well as in the original LPA analysis of the stimulus-evoked LFP data from rat barrel cortex (Einevoll et al., 2007).

The fitted time constants of the two postynaptic LFP contributions are similar: τ_1_ = 3.85 ms and τ_2_ = 4.15 ms, respectively (This is also similar to the fitted time constant τ_1_ = 5.32 ms found with gLPA-1). The fitted delay parameters are different, however. While the delay of the first component is Δ_1_ = 0.48 ms (similar to the value Δ_1_ = 0.40 ms found for gLPA-1), the second component starts more than four milliseconds later, i.e., Δ_2_ = 4.80 ms.

#### 3.2.3. gLPA-3: three postsynaptic LFP profiles

**Figure 4:**
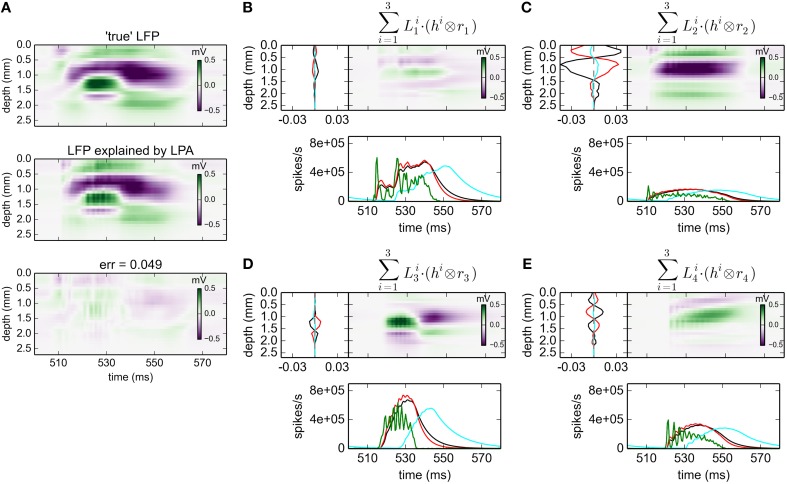
**gLPA-3 decomposition of LFP data**. Results from fitting gLPA-3 (*K* = 3; Equation 4) to LFP data for a time segment where the network is driven by 12.5 Hz oscillatory input in the thalamic relay cells. **(A)** Comparison of ground-truth LFP (“true” LFP; top) with LPA prediction (middle) with difference shown in bottom panel. **(B–E)** Estimated contributions from the four laminar components (color plots) with spatial profiles (black:$L_{n}^{1}$, red:$L_{n}^{2}$, cyan:$L_{n}^{3}$) shown left and temporal profiles [green:*r*_*n*_(*t*); black:$R_{n}^{1}\left(t\right)=h^{1}⊗r_{n}$, red:$R_{n}^{2}\left(t\right)=h^{2}⊗r_{n}$, cyan:$R_{n}^{3}\left(t\right)=h^{3}⊗r_{n}$] below. Fitted parameters: Δ_1_ = 0.02 ms, τ_1_ = 6.85 ms, Δ_2_ = 0.05 ms, τ_2_ = 4.70 ms, Δ_3_ = 10.34 ms, τ_3_ = 12.01 ms.

With three postynaptic LFP profiles, i.e., gLPA-3, the relative error is reduced further from 0.093 for gLPA-1 and 0.059 for gLPA-2, to 0.049 (Figure 4; For the depicted time window only, the error is 0.056). Thus, the relative reduction in error by going from two (gLPA-2) to three (gLPA-3) postsynaptic LFP contributions is smaller (17%) than when going from one (gLPA-1) to two (gLPA-2) contributions (37%). We also observe that the addition of a third LFP kernel in gLPA-3 does not simply add a new component compared to the two components identified by gLPA-2. The two fitted components in gLPA-2 had similar time constants τ_1_ and τ_2_ of about 4 ms, but delays Δ_1_ and Δ_2_ differing by more than 4 ms. The three fitted components in gLPA-3, on the other hand, have three rather different time constants (τ_1_ = 6.85 ms, τ_2_ = 4.70 ms, τ_3_ = 12.01 ms), and delays Δ varying from 0 to more than 10 ms.

### 3.3. Interpretation of gLPA components

Proper assessment of the performance of data analysis methods is much easier when benchmarking data for which the ground truth is known, is available (Denker et al., 2012). In the context of laminar population analysis this will mean benchmarking data where the postsynaptic LFP contribution following action-potential firing in a specific laminar population is available. While such benchmarking data are very difficult, if possible at all, to come by by experimental means, they can straightforwardly be constructed in the present model world.

**Figure 5:**
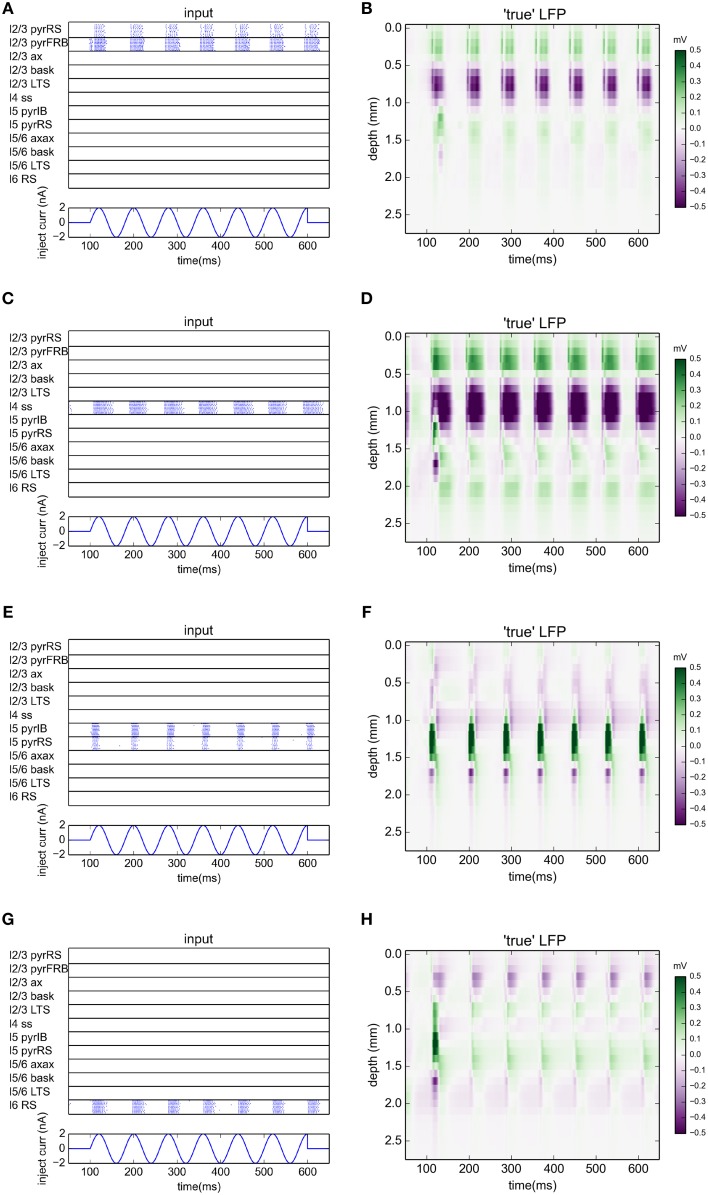
**Ground-truth LFPs activated by single laminar populations**. **(A,C,E,G)** Raster plots of network activity from excitatory populations used in gLPA analysis for simulation with oscillatory current with frequency 12.5 Hz injected to the thalamus for times between 100 and 600 ms. **(A)** layer 2/3 (*n* = 1), **(B)** layer 4 (*n* = 2), **(C)** layer 5 (*n* = 3), **(D)** layer 6 (*n* = 4). **(B,D,F,H)** Corresponding LFP profiles from synaptic activation of an otherwise disconnected network with spikes from individual populations (left column).

The benchmarking data was constructed in two steps. First, the spike times from each excitatory population were recorded from the normal network simulations. Results for the case with a 12.5 Hz stimulation are shown in the panels in the left column of Figure 5. Second, the spikes from each populations were used to generate synaptic inputs (and thus LFPs) onto their postsynaptic targets in an otherwise disconnected network. The resulting LFPs are shown in the four panels in the rightmost column of Figure 5. These spatiotemporal LFP patterns, each reflecting the LFP contribution from a particular laminar population, can then be compared with estimates from gLPA decompositions of the LFP data stemming from the full simulations, cf. Figures 2–4.

**Figure 6:**
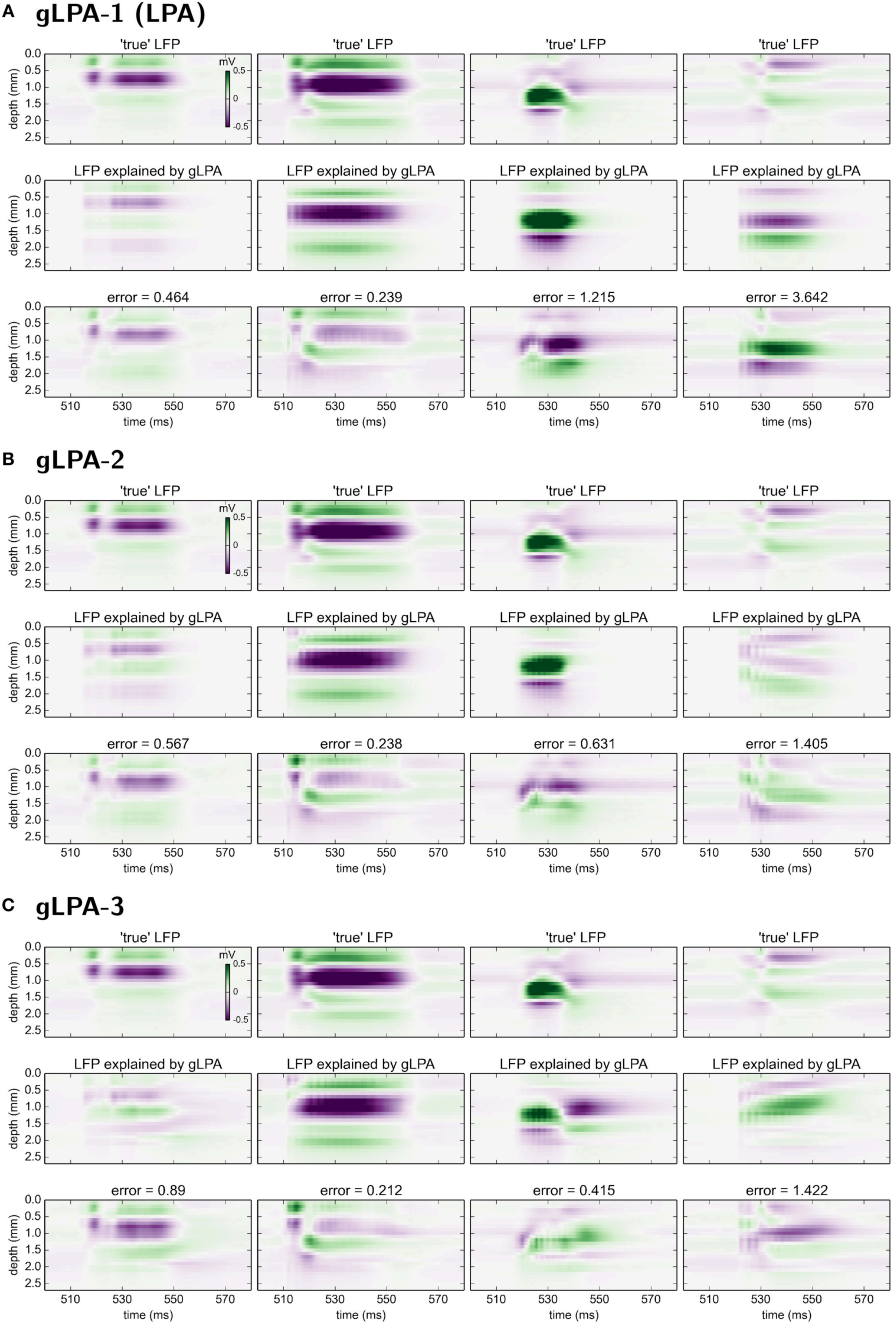
**Comparison of gLPA components with “ground-truth” LFP components from disconnected network**. Top panels: Ground-truth LFP (“true-LFP”) from selective synaptic activation by recorded spikes in neurons belonging to a particular laminar population, to an otherwise disconnected network. Middle panels: Corresponding gLPA components. Bottom panels: Difference between ground-truth (top) and gLPA prediction (middle) panels. First column corresponds to layer 2/3 (*n* = 1), second column to layer 4 (*n* = 2), third column to layer 5 (*n* = 3), fourth column to layer 6 (*n* = 4). **(A)** Top section of 12 panels corresponds to gLPA-1, **(B)** middle section to gLPA-2, and **(C)** bottom section to gLPA-3. Stimulus and time segment correspond to Figures 2–4. The origin of the inversion of the LFP pattern around 540 ms is probably due to afterhyperpolarization of the layer-5 neurons following the spiking (Buzsáki, 1988).

In Figure 6 we compare the predicted spatiotemporal LFP components generated by the various gLPA methods with the LFP found by single-population activation of the disconnected network (“true” LFP). The best agreement is observed for the layer-4 population (*n* = 2, second column in Figure 6) where visual comparison reveals very similar patterns and a relative mean square deviation < 24 for all three gLPA methods (Note that only the 12.5 Hz data is included in this error measure). The predictions for the layer-5 population (*n* = 3, third column) are also fairly accurate both for gLPA-2 and gLPA-3, with gLPA-3 also picking up the abrupt “inversion” of the LFP pattern around *t* = 540 ms which is probably due to afterhyperpolarization of the layer-5 neurons following the spiking (Buzsáki, 1988). gLPA-2 predicts a qualitatively accurate postsynaptic LFP pattern for the layer-2/3 population (*n* = 1, 1. column in Figure 6), while gLPA-3 makes a poorer prediction for this population. For the layer-6 population all gLPA methods essentially fail in predicting accurate postsynaptic LFP patterns.

**Figure 7:**
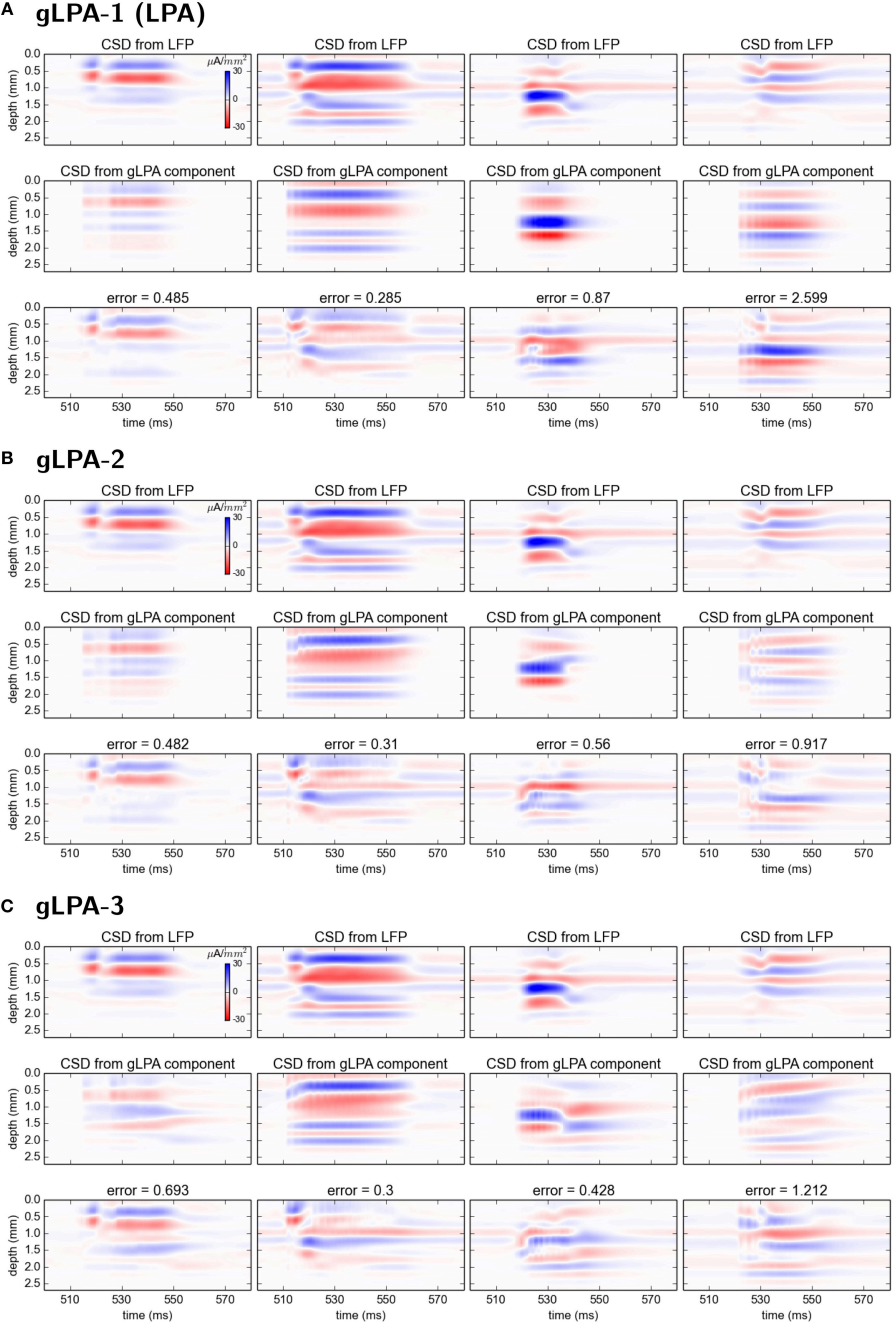
**Comparison of CSD profiles from gLPA components with profiles from “ground-truth” LFP components from disconnected network**. **(A–C)** CSD components computed from the LFP components in Figures 6(A–C) by means of the kCSD method (see Methods).

Comparison of the corresponding CSD patterns is shown in Figure 7, revealing that both gLPA-2 and gLPA-3 in general predict quite accurate spatiotemporal CSD components for the layer 2/3, layer-4, and layer-5 populations, while they largely fail for the layer-6 population.

### 3.4. gLPA decomposition with population firing rates estimated from MUA

So far we have taken advantage of being in a simulated model world where every spike, in fact every minute detail of the system, can be tracked. This has allowed us to investigate the second step of gLPA analysis, i.e., the estimation of LFP profiles given a set of time-resolved population firing rates *r*_*n*_(*t*), in detail. When analysing experimental data, these population firing rates must instead be estimated from the data itself.

Population firing rates may be estimated from experiments in several ways. Multielectrodes with small electrode contacts record sharp spikes from neurons close to the contacts (Buzsáki, 2004). These can detected from MUA recordings by some thresholding procedure and pooled to provide estimates of population firing rates. In the original LPA analysis of Einevoll et al. (2007) the multielectrode contacs were relatively large (40 μm in diameter) and as a result sharp spikes were less prominent, presumably due to spatial averaging effects (Ness et al., 2015). Thus, the population firing rates were instead estimated by rectification of the high-pass filtered extracellular signals, a procedure that has been shown to give results similar to the spike-detection method in an experimental setting (Ulbert et al., 2001) and to ground-truth spike-train results in a detailed model study (Pettersen et al., 2008). While a comprehensive study of the applicability and accuracy of the procedures for estimating population firing rates are beyond the scope of the present study, we here briefly investigate the effect on the estimated LFP profiles when replacing ground-truth population firing rates with population firing rates estimated from MUA signals.

**Figure 8:**
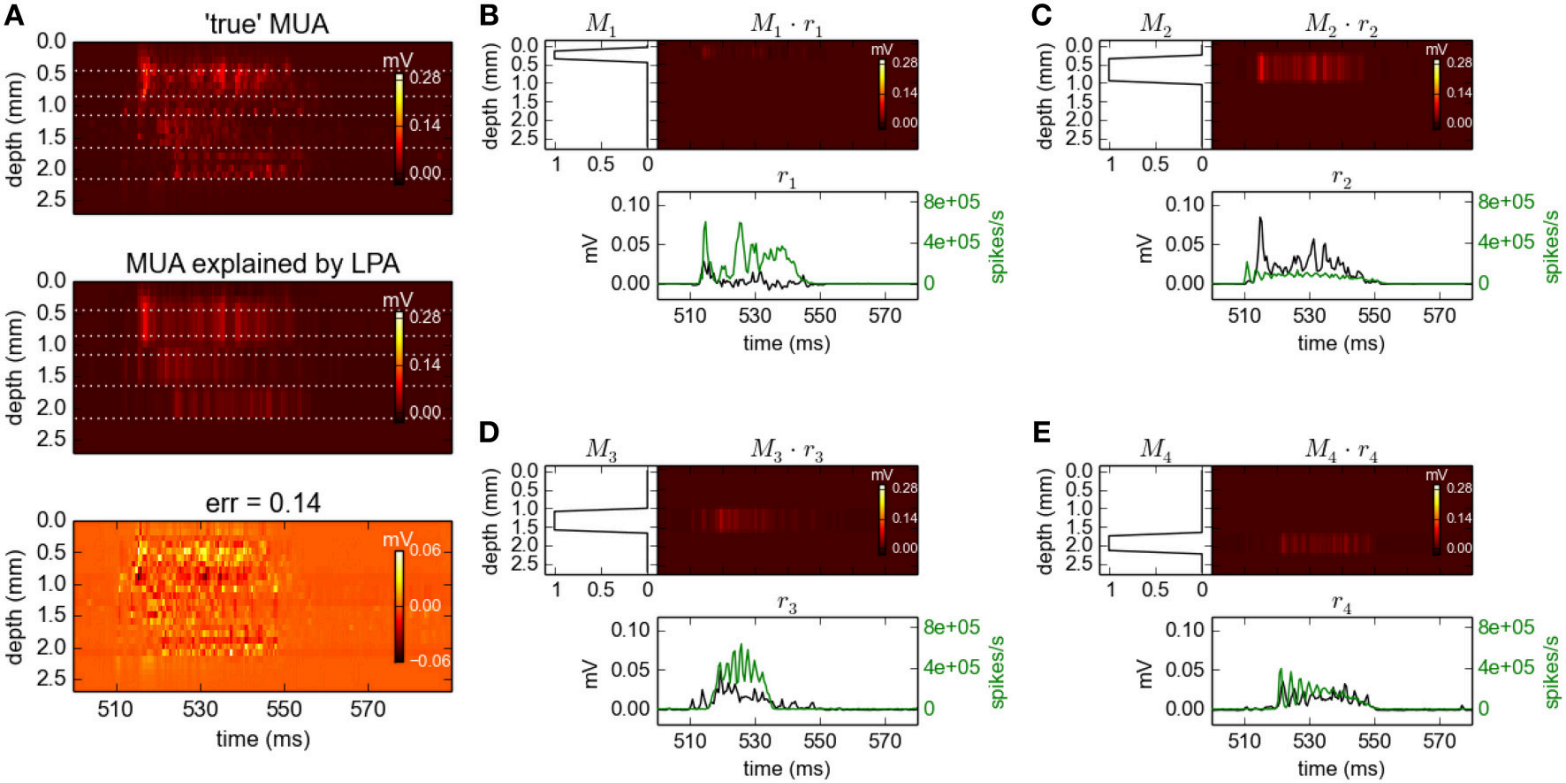
**Population decomposition of MUA signals**. **(A)** Comparison of ground-truth MUA (“true' MUA; top) with MUA signal predicted by best fit to MUA decomposition model in Equation 9 (middle) with difference shown in bottom panel. Data corresponds to the time segment where the network is driven by 12.5 Hz oscillatory input from the thalamic relay cells as in Figure 2. Mean square error of the predicted MUA across the entire simulation protocol is 0.14. **(B–E)** Estimated contributions to the MUA signal from the four laminar components (color plots) with spatial profiles (*M*_*n*_(*z*)) shown left. Population firing rates [black:*r*_*n*_(*t*)] are shown below together with corresponding ground-truth firing rates used in previous gLPA analysis (green). Note the different units for the two firing-rate estimates as the MUA-based estimates (black) do not predict absolute magnitudes of firing rates, only the relative time course (see text).

In the topmost panel in Figure 8A we see the high-pass filtered and subsequently rectified MUA for the same time slot as considered above for the LFP signals. In the panel immediately below we see the best fit of Equation 9 when assuming *N*_*pop*_ = 4 populations in the optimization, illustrating that the MUA model accounts well for the MUA data. The corresponding fitted spatial (*M*_*n*_(*z*)) and temporal profiles [*r*_*n*_(*t*), black lines] are shown in Figure 8B. Note that these MUA-based estimates for the population firing rates (*r*_*n*_(*t*)) do not predict absolute magnitudes of firing rates, only the relative time course (Einevoll et al., 2007; Pettersen et al., 2008).

Comparison with the ground-truth firing rates (green lines) reveals some interesting differences. First, the estimated firing rate for population 1 (*r*_1_(*t*)) is seen to be in poor agreement with the ground-truth firing rate of population 1. However, the estimated firing rate for population 2 (*r*_2_(*t*)) is instead seen to be in good agreement with the population-1 ground-truth rate. Inspection of the estimated spatial profiles *M*_1_ and *M*_2_ explains why: While the excitatory neurons of layer 2/3 (1000 RS and 50 FRB cells with somas at depths 450–850 μm, cf. Table 1) and layer 4 (240 SS cells with somas at depths 850–1150 μm) were assumed to be separate laminar populations in the above gLPA-analysis using the ground-truth firing-rates, these populations have been effectively merged in the MUA-based analysis. This is not surprising since there are only 240 SS cells compared to 1050 RS+FRB cells, so that the spike signals from the population of layer-4 SS cells are evidently too small to be identified as a separate laminar population. However, the *sum* of the population firing rates of populations 1 and 2 are seen to be quite well estimated from the MUA signal. The depth profiles of the layer-5 (800 IB and 200 RS cells with somas at depths 1150–1650 μm, cf. Table 1) and layer-6 populations (500 RS cells with somas at depths 1650–2150 μm) are identified quite accurately by the MUA-based analysis, cf. *M*_3_(*z*) and *M*_4_(*z*) in Figure 8B. The depicted population firing rates of these populations only reproduce the overall gross features of the temporal firing-rate profiles, though.

If the goal *per se* is to estimate the time-resolved population firing rates as accurate as possible, the above comparisons point to further explorations of questions such as whether, say, three populations (*N*_*pop*_ = 3) should be assumed instead of four, whether the MUA-estimated firing rates is better compared with ground-truth population firing rates where also inhibitory neurons are included, or whether spatial profiles *M*_*n*_(*z*) with other shapes than trapezoids should be considered. However, the key question here is how much the LFP profiles predicted by gLPA is affected by inaccuracies in the estimated population firing rates.

**Figure 9:**
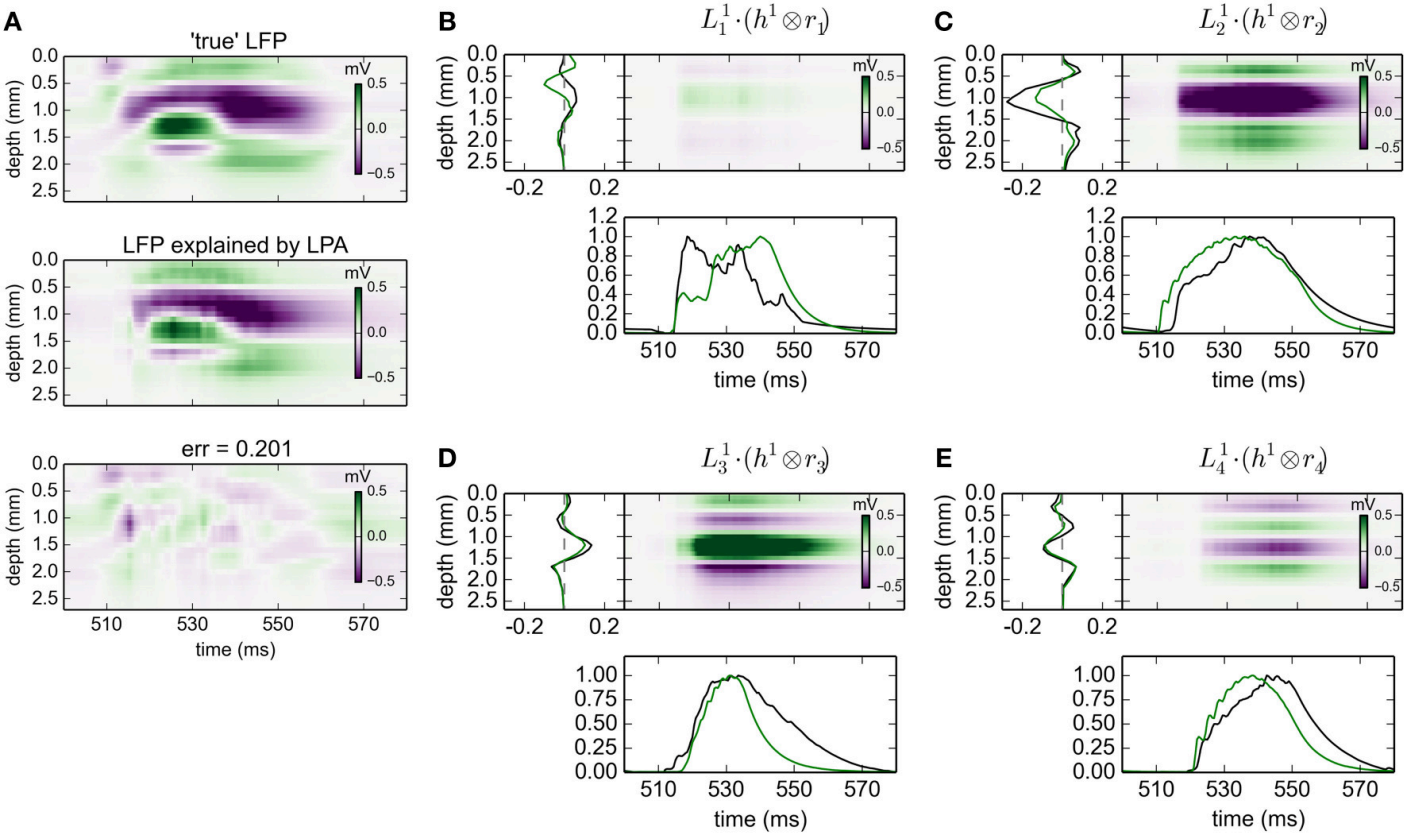
**Population decomposition of LFP signals with MUA-based firing-rate estimates**. Results from fitting gLPA-1 (*K* = 1; Equation 2) to LFP data for a time segment where the network is driven by 12.5 Hz oscillatory input in the thalamic relay cells with MUA-based firing-rate estimates, cf. Figure 8. **(A)** Comparison of ground-truth LFP (“true” LFP; top) with LPA prediction (middle) with difference shown in bottom panel. **(B–E)** Estimated contributions from the four laminar components (color plots) with spatial profiles ($L_{n}^{1}$) shown left and temporal profiles [black:$R_{n}\left(t\right)=h^{1}⊗r_{n}$] below. Fitted parameters: Δ_1_ = 0.63 ms, τ_1_ = 12.65 ms. To facilitate comparison with LPA decomposition results from using ground-truth firing rates depicted in Figure 2 (green spatial and temporal profiles), the time-convolved firing rates *R*_*n*_(*t*) are scaled to have a maximum value of one in the depicted time interval, while $L_{n}^{1}$ is scaled correspondingly to keep the product $L_{n}^{1}\cdot R_{n}\left(t\right)$ fixed.

The results for gLPA-1, i.e., the original LPA method, when using the MUA-derived population firing rates are depicted in Figure 9. While the relative LFP fitting error is about twice as large as that for the results with ground-truth firing rates in Figure 2 (0.2 vs. 0.09), the fitted spatial profiles $L_{n}^{1}$ are generally very similar. When comparing the spatial profiles for the two methods in Figures 9B–E, we see that they are almost identical for populations 3 ($L_{3}^{1}$) and 4 ($L_{4}^{1}$), and mainly differ by a scaling factor for population 2 ($L_{2}^{1}$). Only for population 1 ($L_{1}^{1}$), the predictions are substantially different, but this population in any case carries very little of the total LFP signal in the resulting fit (cf. the spatiotemporal plot in Figure 9B). Thus, despite the inaccuracies in the prediction of the population firing rates (cf. Figure 8), we find the predicted LFP profiles to overall be quite accurate in the present Traub-model example.

## 4. Discussion

In the present paper we have introduced the *generalized laminar population analysis (gLPA)* for analysis of multielectrode data from cortex, and possibly other brain structures with a laminar organization. gLPA extends the original LPA method (Einevoll et al., 2007) in that it allows for a larger set of basis functions in which the postsynaptic LFP contribution generated by a single population can be expanded. To test the various versions of gLPA, that is, gLPA-1 (original LPA), gLPA-2 and gLPA-3 with one, two, and three separate LFP basis functions, respectively, we have generated model-based benchmarking data. In particular, we have used a biophysical forward-modeling scheme based on volume-conductor theory to compute virtual LFP data from network activity in the comprehensive thalamocortical network model developed by Traub and coworkers (Traub et al., 2005).

We first investigated how much of the LFP data the various gLPA versions could account for. The benchmarking data set was based on oscillatory network activation by means of the thalamic neurons receiving sinusoidal currents for a set of eight different frequencies between 2 and 200 Hz. For the original LPA method (corresponding to gLPA-1) the error, that is, relative mean square deviation between the true LFP and the LPA prediction, was 0.093. For gLPA-2 this was reduced by 37% to 0.059. For gLPA-3 an error of 0.049 was found, implying a more moderate error reduction of only 17% compared to gLPA-2.

While the gLPA methods were all found to account well for the “true” LFP data, that is, the fitting errors were all small, a close inspection of the gLPA-predicted LFP reveals small temporal “ripples” not present in the “true” LFP. These ripples, with ridges typically a couple of milliseconds apart, stem from the similarly rippled temporal structure of the population firing rates (see e.g., green lines in panels D and E in Figure 2) combined with the choice of the sharp onset exponentially decaying temporal kernels *h*(*t*). With temporal kernels with a smoother onset, for example a so-called α-function (Dayan and Abbott, 2001), or a temporally more smoothed firing rate, these ripples would expectedly be reduced.

Both experiments (Einevoll et al., 2007) and modeling studies (Glabska et al., 2014) have revealed transient spatiotemporal LFP patterns following strong synaptic activation that are not separable in space and time, i.e., not describable by a product of single spatial and temporal functions. Such patterns are expected from the so called intrinsic dendritic filtering effect (Pettersen and Einevoll, 2008; Lindén et al., 2010; Pettersen et al., 2012) since high-frequency parts of the LFP originate from shorter current dipoles than the low-frequency parts. gLPA-2 with two postsynaptic (spatiotemporally separable) LFP kernels can account for these effects, while gLPA-1 with a single LFP kernel by construction cannot.

When comparing with ground-truth results for the postsynaptic profiles, gLPA-1, gLPA-2, and gLPA-3 all predict qualitatively correct postsynaptic LFP and corresponding CSD profiles for the three topmost laminar populations (layer 2/3, layer 4, layer 5). The agreement with generally better for CSD than LFP, however. Overall, both gLPA-2 and gLPA-3 are seen to give more accurate results than gLPA-1. The comparison of gLPA-2 vs. gLPA-3 is less clear-cut, and it is difficult to make a clear general recommendation from this study alone regarding what method to use in other situations.

In fact, while we have presented some compelling evidence that the gLPA methods give fairly accurate results, we have not pursued a systematic study of the expected relative merit of the candidate gLPA methods, i.e., gLPA-1, gLPA-2, and gLPA-3, when comparing with experiments. While gLPA-3 gives the smallest fitting errors, it also has the most free parameters and thus the highest risk of overfitting. In this case the prediction accuracy will not improve, or even be reduced despite the better data fit (This was exemplified above by the similar prediction accuracy of postsynaptic CSD and LFP profiles for gLPA-2 and gLPA-3, despite the better fit of gLPA-3 to model data). To select among different candidate models with different number of model parameters, it is customary when comparing to experimental data to augment the present error term with a model-complexity penalty based on the number of free model parameters. This is typically achieved using Akaike (AIC) or Bayesian (BIC) information criteria (Akaike, 1974; Schwartz, 1978). In dynamical causal modeling (DCM) (David and Friston, 2003) where neurophysiological data is fitted to a set of differential equations representing the dynamics of underlying neural-mass models, a more general Bayesian model selection scheme is used (Marreiros et al., 2010). A detailed inquiry into the best gLPA scheme to use in various experimental settings is not pursued here, however.

In this work we have rather concentrated on conceptual aspects of LPA analysis, namely how the addition of components changes the description of the observed LFP signal. In the context of experimental data analysis, however, one must also address the other element of LPA analysis, namely the estimation of population firing rates from data. In an *in vivo* setting such population firing rates may be estimated from the high-frequency part (MUA) of extracellular recordings either by counting spikes detected by a suitable spike-detection method (Ulbert et al., 2001; Buzsáki, 2004) or by rectification of the MUA signal (Ulbert et al., 2001; Einevoll et al., 2007; Pettersen et al., 2008). For the present Traub-model example we found that despite differences in the predicted population firing rates, the spatial LFP profiles predicted from LPA analysis (gLPA-1) were generally similar.

Finally, the present model-based approach for developing and testing methods for LFP analysis is an open-ended approach that should be pursued further as new, even more comprehensive and detailed network models become available (Denker et al., 2012; Einevoll et al., 2013a).

## Author contributions

HG performed simulations and analyzed data. EN implemented gLPA and analyzed data. AD, AMD, GE proposed gLPA method and designed study. DW designed simulations and analysis. HG, GE, DW wrote the article.

### Conflict of interest statement

The authors declare that the research was conducted in the absence of any commercial or financial relationships that could be construed as a potential conflict of interest.
